# Exocrine tissue-driven TFF2 prevents apoptotic cell death of endocrine lineage during pancreas organogenesis

**DOI:** 10.1038/s41598-018-38062-9

**Published:** 2019-02-07

**Authors:** Koji Hirata, Sota Kodama, Yasuhiro Nakano, Yasuko Minaki-Nakagawa, Yoshiki Aoyama, Morito Sakikubo, Toshihiko Goto, Masahiro Yoshida, Toshihiko Masui, Takuya Yamamoto, Shinji Uemoto, Yoshiya Kawaguchi

**Affiliations:** 1 Department of Hepato-Biliary-Pancreatic Surgery and Transplantation, Kyoto University Graduate School of Medicine, Kyoto, Japan; 2 Department of Clinical Application, Center for iPS cell Research and Application, Kyoto, Japan; 3 Department of Life Science Frontiers, Center for iPS Cell Research and Application, Kyoto, Japan

## Abstract

During embryogenesis, exocrine and endocrine pancreatic tissues are formed in distinct regions within the branched ductal structure in mice. We previously reported that exocrine-specific inactivation of *Pdx1* by Elastase-Cre caused not only hypoplastic exocrine formation but also substantial endocrine defects resulting in diabetic phenotype, indicating the existence of an exocrine-driven factor(s) that regulates proper endocrine development. In this study, we identified Trefoil Factor 2 (TFF2) as an exocrine gene expressed from embryonic day 16.5 to adulthood in normal mice but significantly less in our *Pdx1* mutants. Using *in vitro* explant culture of embryonic pancreatic tissue, we demonstrated that TFF2 prevented the apoptosis of insulin-producing cells but that antagonizing CXCR4, a known TFF2 receptor, suppressed this anti-apoptotic effect in the mutants. Furthermore, the antagonist in normal pancreatic tissue accelerated the apoptosis of insulin-producing cells, indicating that the TFF2/CXCR4 axis maintains embryonic insulin-producing cells in normal development. TFF2 also suppressed the apoptosis of Nkx6.1+ endocrine precursors in mutant pancreata, but this effect was unperturbed by the CXCR4 antagonist, suggesting the existence of an unknown receptor for TFF2. These findings suggest TFF2 is a novel exocrine factor that supports the survival of endocrine cells in the multiple stages of organogenesis through distinct receptors.

## Introduction

The adult pancreas plays two roles. One is exocrine function, in which acinar cells secrete digestive enzymes into the duodenum. The other is endocrine function, in which islets secrete hormones into the bloodstream to maintain blood glucose homeostasis. During embryonic organogenesis, both exocrine and endocrine pancreatic tissues originate from the pancreatic buds. Within the pancreatic buds, epithelial cells gradually form the ductal plexus and undergo remodeling to form a branched duct structure composed of a CPA- and Ptf1a-expressing tip domain and a Nkx6.1-positive trunk domain^1^. During segregation of the tip/trunk regions, the differentiation ability of epithelial cells is spatiotemporally regulated; Pdx1^+^Ptf1a^+^cMyc^high^Cpa1^+^ progenitor cells are multipotent at first but lose their ability for endocrine differentiation after E13-14, whereas Nkx6.1+ cells in the trunk region can differentiate into endocrine and duct cells^1,2^. In endocrine lineage, Ngn3+ endocrine precursor cells bud out from the lining of the Nkx6.1+ ductal trunk and differentiate into all cell types of the islet, including glucagon+ α cells, insulin+ β cells, somatostatin+ δ cells and pancreatic polypeptide+ PP cells.

The necessity of exocrine tissue formation for proper endocrine development was assessed in our previous study by using *Elastase-Cre;Pdx1*^*loxP/loxP*^ (Pdx1cKO) mice, in which Pancreatic and duodenal homeobox 1 (*Pdx1*), an indispensable gene for pancreas organogenesis, can be inactivated specifically in exocrine tissue. We demonstrated that the mutant mice not only showed hypoplastic formation of exocrine tissue, but also severe endocrine defects during embryogenesis characterized by accelerated apoptosis in the trunk region leading to reduced numbers of Nkx6.1+ cells, Ngn3+ endocrine precursors and Insulin+ cells at embryonic day 14.5 (E14.5). Moreover, the postnatal expansion of endocrine cells was extremely poor in the mutants, resulting in glucose intolerance at postnatal day 28 (P28). These observations suggested the existence of essential factors in the exocrine tissue that regulate proper development and proper function at multiple differentiation steps of endocrine lineage^3^.

In the present study, we aimed to identify the responsible factor(s). We performed microarray analyses and identified Trefoil Factor 2 (TFF2) as an exocrine factor in embryonic and adult pancreas in normal mice, but found its expression was extremely low in Pdx1cKO mice. Our explant culture experiments using embryonic pancreatic tissue demonstrated that TFF2 functions to protect endocrine cells against apoptosis at multiple differentiation stages including Nkx6.1+ endocrine progenitors and Insulin+ cells. These findings suggest TFF2 is a novel exocrine factor that acts as a paracrine signal and is required for proper endocrine formation during pancreas organogenesis.

## Results

### TFF2 is expressed in pancreatic exocrine tissue

To identify factors that are expressed in exocrine pancreas tissue and regulate proper endocrine development, we first compared the gene expression profiles of pancreatic tissue at P1 of control mice and Pdx1cKO mice. As shown in Supplementary Table S1, microarray analysis revealed that the expressions of many digestive enzymes including Ctrl (chymotrypsin-like-peptide) and Pnliprp2 (pancreatic lipase-related protein 2) were significantly reduced in Pdx1cKO pancreata, which is consistent with the severely hypoplastic exocrine tissue of these mice. Among the genes showing reduced expression, we focused on Trefoil factor 2 (TFF2), which was previously reported to stimulate the proliferation of β cells in adult pancreas^4^, as a candidate factor responsible for the endocrine defects. A reduction of *TFF2* mRNA expression in mutant pancreata at P1 was confirmed by RT-PCR analysis (Supplementary Fig. S1A). As for other genes of the TFF family, qPCR analyses showed similar expression levels of *TFF1* mRNA and *TFF3* mRNA in Pdx1cKO and control pancreata at P1 (Supplementary Fig. S1B).

**Figure 1:**
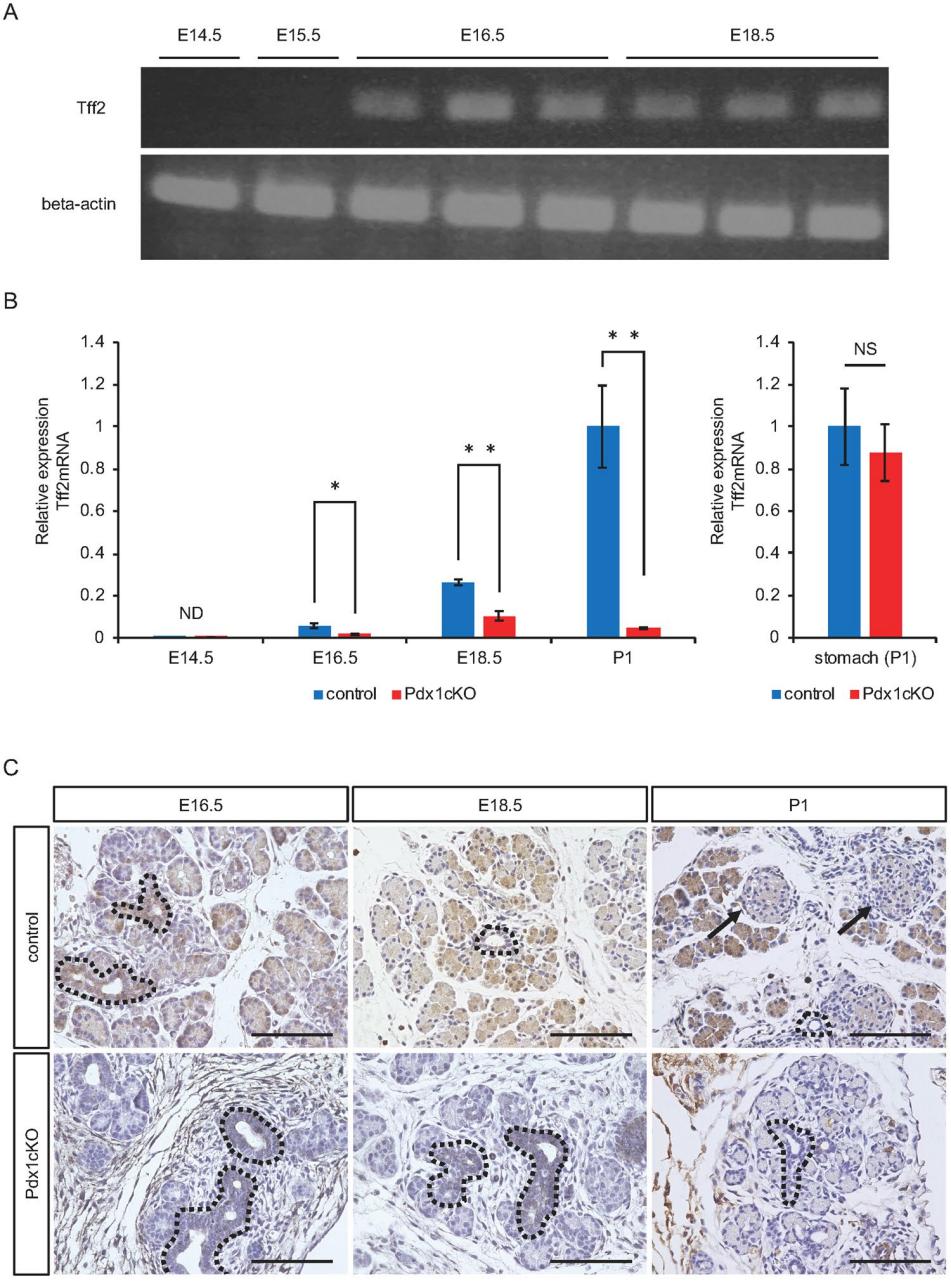
Elastase-Cre-mediated Pdx1 inactivation reduces acinar TFF2 in embryonic and neonatal pancreas. (**A**) The expression of *TFF2* was detected by RT-PCR in control mice pancreas from E16.5. The original data are shown in Supplementary Fig. S1C. (**B**) Expression of *TFF2* is significantly less in Pdx1cKO mice (red) than in control mice (blue). (control mice: n = 7 at E14.5, n = 5 at E16.5, n = 5 at E18.5, and n = 7 at P1; Pdx1cKO mice: n = 5 at E14.5, n = 6 at E16.5, n = 6 at E18.5, and n = 7 at P1; p = N.D at E14.5, p = 0.041 at E16.5, p = 0.0065 at E18.5 and p = 0.0040 at P1). Note that the expression of *TFF2* in the mutant stomach is equivalent to that in control stomach at P1 (right panel) (control mice, n = 3, Pdx1cKOmice, n = 3, p = 0.68122). (**C**) Immunostaining of TFF2. TFF2 expression was detected in exocrine cells including the proximal (dotted lines) and distal ducts and acinar cells, but not in islets (arrows) in control mice (upper panels). In Pdx1cKO mice, TFF2 expression was hardly detectable except in the proximal ducts (dotted lines), which were not recombined by Elastase-Cre (bottom panels). These expression patterns were confirmed in at least three individual mice for both genotypes. Scale bars, 100 μm. Bars represent the mean value ± SE. *p < 0.05, **p < 0.01.

Next, we analyzed the expression pattern of TFF2 in the pancreas. During normal pancreatic development, *TFF2* mRNA was first expressed at E16.5 and increased as development proceeded (Fig. 1A,B). On the contrary, although *TFF2* mRNA in the Pdx1cKO pancreata was also first expressed at E16.5, the expression was much lower and it did not tend to increase with time (Fig. 1B). In normal mice, immunohistochemistry detected TFF2 expression in the proximal and distal ductal structures and in developing acinar cells at E16.5 (Fig. 1C). At E18.5, however, while most acinar cells still expressed TFF2, the expression in the proximal ducts (trunk region) was reduced. Finally, strong immunostaining of TFF2 was maintained in acinar cells, but was almost undetectable in islets at P1. In Pdx1cKO mice, TFF2 was hardly detectable at any of the three stages except in proximal ducts, which were not affected by the Elastase-Cre recombination (Fig. 1C). Interestingly, *in situ* hybridization demonstrated acinar-specific expression of *TFF2* mRNA in adult pancreas (Supplementary Fig. S2), which is inconsistent with a previous report that showed TFF2 expression in adult islets by immunochemistry^4^. Based on our findings, we concluded that TFF2 is expressed in normal embryonic and adult pancreatic exocrine tissue, but significantly suppressed in the same tissue of Pdx1cKO mutants.

### Accelerated apoptosis of embryonic Nkx6.1+ trunk cells and Insulin+ cells in Pdx1cKO mutant pancreata

**Figure 2:**
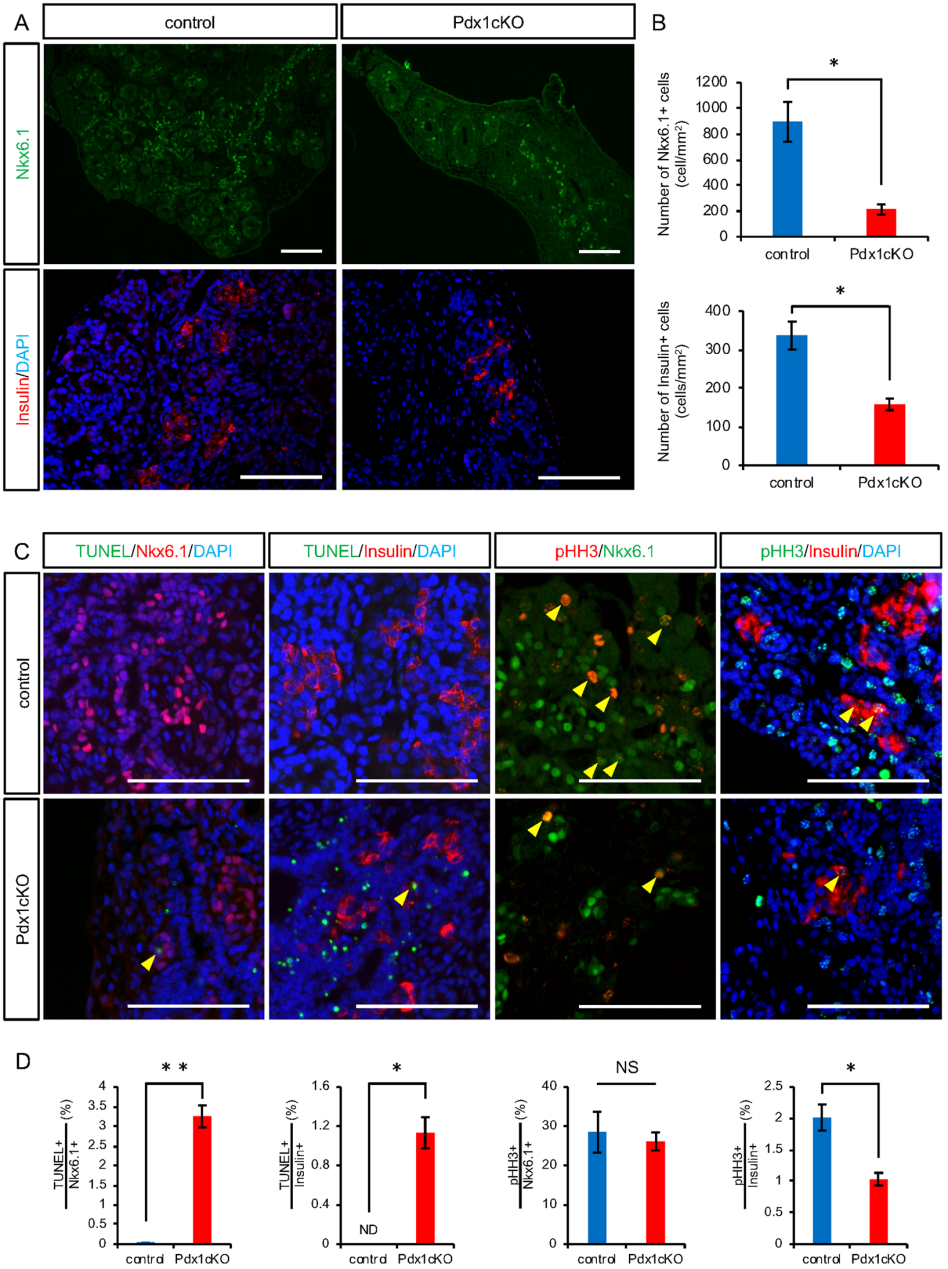
Reduced number and accelerated apoptosis of Nkx6.1-positive cells and insulin-producing cells at E16.5 in Pdx1cKO mice. (**A**) Immunostaining of Nkx6.1 (green), insulin (red) and DAPI (blue) in control (left panels) and Pdx1cKO (right panels) pancreata at E16.5. (**B**) Quantitative analysis revealed that the numbers of Nkx6.1+ cells (upper panel) and Insulin+ cells (bottom panel) in Pdx1cKO mice (red) are significantly less than in control mice (blue) at E16.5. (**C**) TUNEL and pHH3 analyses of E16.5 pancreata. Yellow arrowheads show TUNEL- or pHH3-positive cells in Nkx6.1+ or Insulin+ cells. (**D**) Quantitative analyses show that the apoptosis rates of Nkx6.1+ cells and Insulin+ cells were significantly higher in Pdx1cKO pancreata (red) compared to control pancreata (blue) (left two panels). The proliferation rate of Nkx6.1+ cells was equivalent, but that of Insulin+ cells was less in Pdx1cKO pancreata (right two panels). Scale bars, 100 μm. Bars represent the mean ± SE. *p < 0.05, **p < 0.01.

Since the onset of TFF2 expression began at E16.5, we analyzed the endocrine phenotype of Pdx1cKO mice at this stage. We found that the numbers of Nkx6.1+ trunk cells, which are the origin of endocrine lineage, and Insulin+ cells were significantly reduced in mutants compared with control mice (Nkx6.1+ trunk cells: control, 894.4 ± 155.1 cells (n = 5), Pdx1cKO, 213.0 ± 41.1 cells (n = 5), p = 0.015; Insulin+ cells: control, 337.0 ± 36.6 cells (n = 3), Pdx1cKO, 158.3 ± 14.7 cells (n = 3), p = 0.042) (Fig. 2A,B). To account for this difference, we found accelerated apoptosis of Nkx6.1+ trunk cells and Insulin+ cells in the mutant mice (TUNEL-positive Nkx6.1+ trunk cells: control, 0.02 ± 0.01% (n = 4), Pdx1cKO, 3.25 ± 0.28% (n = 4), p = 0.002; TUNEL-positive Insulin+ cells: control, not detected (n = 3), Pdx1cKO, 1.13 ± 0.16% (n = 3), p = 0.028). In addition, while the proliferation rate of Nkx6.1+ trunk cells in mutant mice was almost the same as in control mice (pHH3-positive Nkx6.1+ trunk cells: control, 28.49 ± 5.21% (n = 5), Pdx1cKO, 26.09 ± 2.32% (n = 5), p = 0.21), that of Insulin+ cells was significantly less (pHH3-positive Insulin+ cells: control, 2.01 ± 0.21% (n = 3), Pdx1cKO, 1.03 ± 0.10% (n = 3), p = 0.043) (Fig. 2C,D).

### TFF2 prevents apoptosis of embryonic insulin-expressing cells through CXCR4

**Figure 3:**
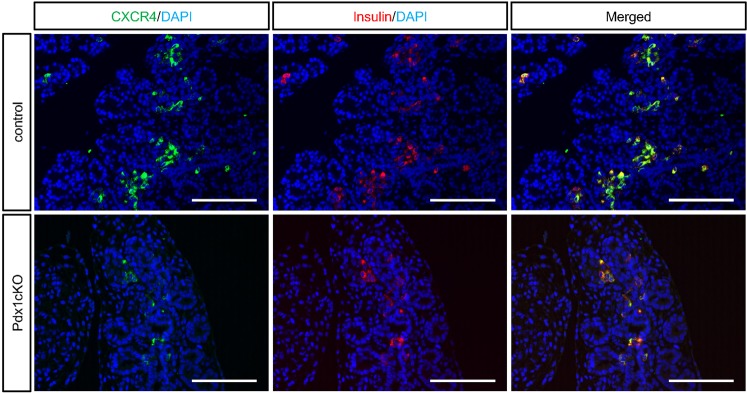
Expression of CXCR4 in insulin-expressing cells. Immunostaining of CXCR4 (green), insulin (red) and DAPI (blue) in control (upper panels) and Pdx1cKO (bottom panels) mice at E16.5. Note that CXCR4 expression is restricted to Insulin+ cells in both mice. Scale bars, 100 μm.

Because previous studies identified CXCR4 as a functional receptor for TFF2 in β cell lines and submandibular gland^5,6^, we analyzed CXCR4 expression in embryonic pancreas. Our immunohistochemical analyses revealed that CXCR4 is restrictively expressed in Insulin+ cells at E16.5 in both control and mutant pancreata (Fig. 3).

**Figure 4:**
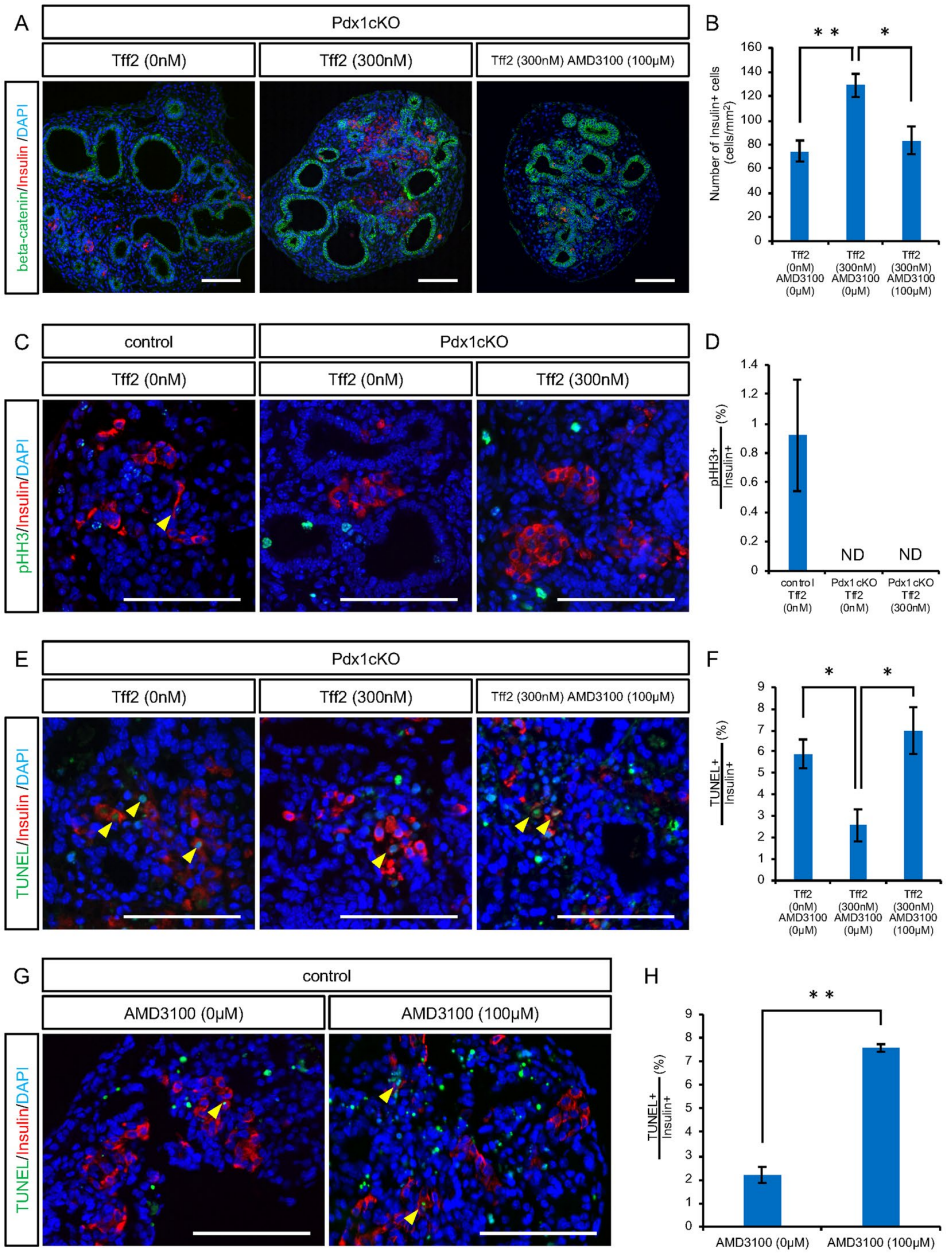
TFF2 prevents apoptosis of embryonic insulin-expressing cells through CXCR4. Explant culture of E16.5 pancreatic tissue for 48 hours. (**A**) Immunostaining of insulin (red), beta-catenin (green) and DAPI (blue) for the cultured explant without reagents (left panel), with rTFF2 (middle panel) and with rTFF2 plus AMD3100 (right panel). (**B**) Quantitative analysis of the number of Insulin+ cells. (**C**) Immunostaining of pHH3 (green), insulin (red) and DAPI (blue) for cultured control explant (left panel) and for Pdx1cKO explant without and with rTFF2 (middle and right panels, respectively). The yellow arrowhead shows pHH3-positive Insulin+ cells. (**D**) Quantitative analyses of Insulin+ cell proliferation. (**E**) Immunostaining of TUNEL (green), insulin (red) and DAPI (blue) for cultured Pdx1cKO explant without reagents (left panel), with rTFF2 (middle panel) and with rTFF2 plus AMD3100 (right panel). Yellow arrowheads show TUNEL-positive Insulin+ cells. (**F**) Quantitative analysis of Insulin+ cell apoptosis in Pdx1cKO explant. In control mice pancreas, CXCR4 protected Insulin+ cells from apoptosis. (**G**) Immunostaining of TUNEL (green), insulin (red) and DAPI (blue) for the control explant without and with AMD3100 (left and right panels, respectively). Yellow arrowheads show TUNEL-positive Insulin+ cells. (**H**) Quantitative analyses of Insulin+ cell apoptosis. Scale bars, 100 μm. Bars represent the mean value ± SE. *p < 0.05, **p < 0.01.

To test the hypothesis that exocrine-driven TFF2 regulates endocrine development, we performed explant culture of pancreatic tissue at E16.5. In our culture condition, the exocrine and endocrine components were kept for 48 hours with dilatation of the pancreatic ducts (Supplementary Fig. S3). We found that the addition of mouse recombinant TFF2 (rTFF2) increased the number of Insulin+ cells in Pdx1cKO pancreata (no reagents, 74.6 ± 8.8 cells (n = 7), rTFF2 300 nM, 128.9 ± 9.6 cells (n = 7), p = 0.0041), and that this elevation was antagonized by the addition of AMD3100^7^, a specific inhibitor of CXCR4 (rTFF2 300 nM + AMD3100 100 μM, 83.5 ± 11.4 cells (n = 4), p = 0.037) (Fig. 4A,B). While TFF2 did not stimulate the proliferation of Insulin+ cells in Pdx1cKO pancreata (pHH3-positive Insulin+ cells: control without reagents, 0.92 ± 0.38% (n = 3), Pdx1cKO without reagents, not detected (n = 4), Pdx1cKO with rTFF2 300 nM, not detected (n = 5)) (Fig. 4C,D), the TFF2/CXCR4 axis prevented their apoptosis. Indeed, our TUNEL staining analyses revealed that TFF2 reduces the percentage of TUNEL + cells among Insulin+ cells in the explant culture of Pdx1cKO pancreata and that this reduction was negated by ADM3100 treatment (no reagents, 5.89 ± 0.67% (n = 3), rTFF2 300 nM, 2.57 ± 0.75% (n = 7), rTFF2 300 nM + AMD3100 100 μM, 6.99 ± 1.08% (n = 4), p = 0.046 (no reagents vs. rTFF2), p = 0.018 (rTFF2 vs. rTFF2 + AMD3100)) (Fig. 4E,F). The anti-apoptotic effect of the TFF2/CXCR4 axis was confirmed by the explant culture of normal pancreata, in which we found that the percentage of TUNEL-positive cells among Insulin+ cells was significantly increased by AMD3100 treatment (no reagents, 2.19 ± 0.34% (n = 3), AMD3100 100 μM, 7.58 ± 0.16% (n = 3), p = 0.0017) (Fig. 4G,H). Taken together, TFF2 expressed in exocrine cells from E16.5 functions to preserve embryonic Insulin+ cells via CXCR4 in normal development. In Pdx1cKO mice, on the other hand, the poor TFF2 expression caused accelerated apoptosis of embryonic Insulin+ cells, which resulted in fewer Insulin+ cells and ultimately the diabetic phenotype after birth.

### TFF2 has an anti-apoptotic effect on Nkx6.1+ trunk cells through an unknown receptor in Pdx1cKO pancreata

**Figure 5:**
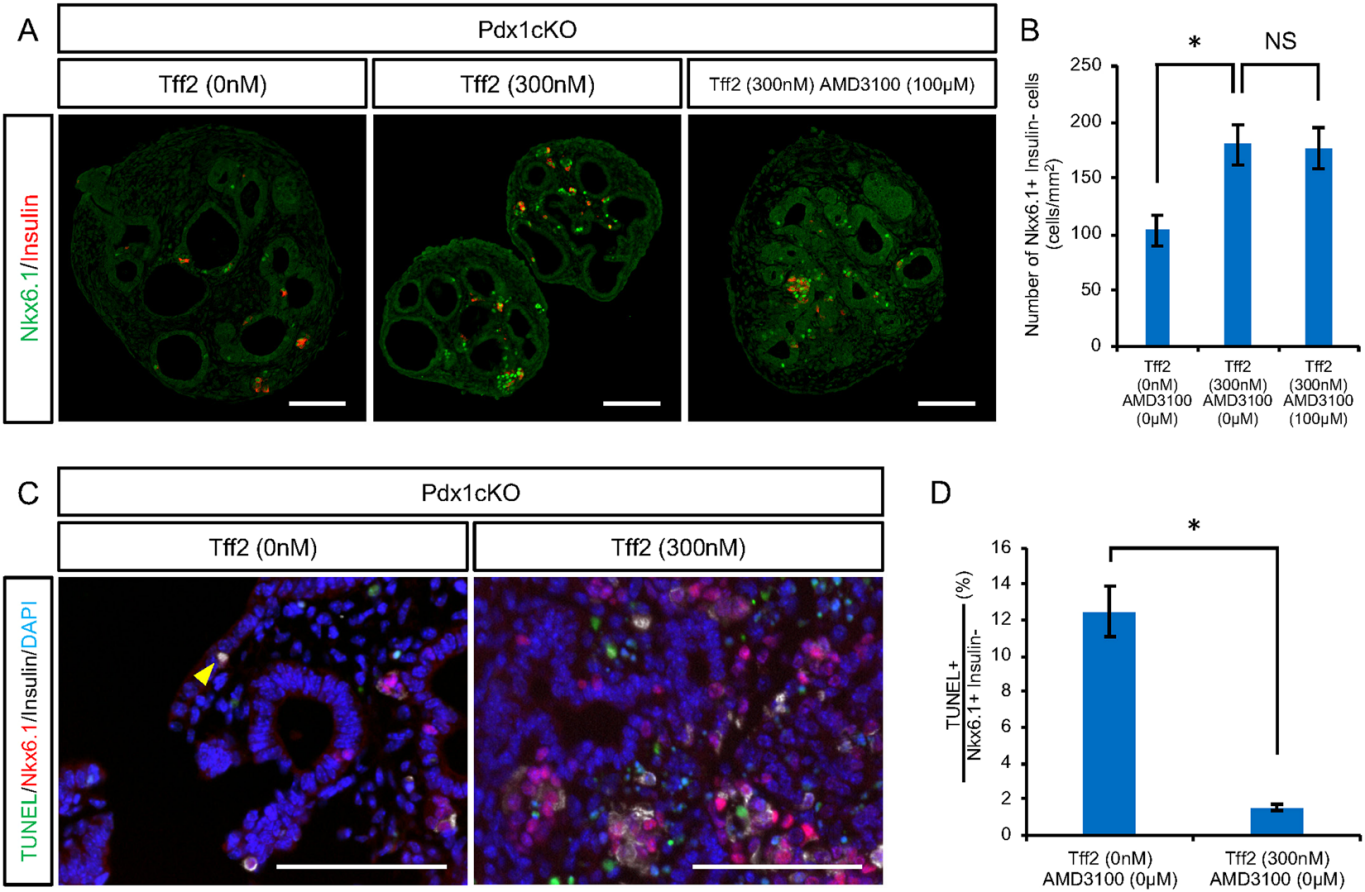
Nkx6.1-positive trunk cells are affected by TFF2. Analyses of Nkx6.1+/Insulin- trunk cells in explant culture experiments. (**A**) Immunostaining of Nkx6.1 (green) and insulin (red) for cultured Pdx1cKO explant without reagents (left panel), with rTFF2 (middle panel) and with rTFF2 plus AMD3100 (right panel). (**B**) Quantitative analyses of the number of Nkx6.1+/Insulin- trunk cells. (**C**) TUNEL staining of cultured Pdx1cKO explant without and with rTFF2 (left and right panels, respectively). (**D**) Quantitative analyses of Nkx6.1+/Insulin- trunk cell apoptosis. Scale bars, 100 μm. Bars represent the mean value ± SE. *p < 0.05.

As mentioned above, Pdx1cKO mice had significantly fewer Nkx6.1+ cells than control mice at E16.5 along with accelerated apoptosis (Fig. 2). In the tissue explant culture experiments, we noticed that TFF2 significantly increased the number of Nkx6.1+/Insulin- trunk cells in mutant pancreata, but that the addition of AMD3100 did not affect this elevation, which is consistent with the undetectable CXCR4 expression in this cell type (no reagents, 103.3 ± 13.7 cells (n = 4), rTFF2 300 nM, 179.8 ± 18.1 cells (n = 5), rTFF2 300 nM + AMD3100 100 μM, 176.7 ± 18.4 cells (n = 3), p = 0.044 (no reagents vs. rTFF2), p = 0.99 (rTFF2 vs. rTFF2 + AMD3100)) (Fig. 5A,B). Again, TFF2 had an anti-apoptotic effect on Nkx6.1+/Insulin- trunk cells, as shown by the reduced percentage of TUNEL + cells (no reagents, 12.10 ± 1.40% (n = 3), rTFF2 300 nM, 1.55 ± 0.18% (n = 3), p = 0.022) (Fig. 5C,D). Therefore, we concluded that TFF2 functions to preserve Nkx6.1+ endocrine precursor cells through an unknown receptor in Pdx1cKO pancreata.

## Discussion

Rising evidence supports the notion that exocrine-driven factors regulate endocrine islets in adult pancreas. Evidence includes reports that found Reg1 stimulates β cell proliferation in a rat regeneration model^8^, exocrine tissue extracts in a pancreatic-duct-ligated model (PDL model) induce β cell de-differentiation *in vitro*^9^, and Sostdc1 functions to orchestrate insulin secretion and glucose homeostasis under metabolic stress^10^. These factors are activated in response to tissue injury or metabolic stress of the adult pancreas, but the molecular basis of the exocrine-endocrine interaction during embryonic organogenesis is unknown. In the present study, we identified TFF2 as a novel exocrine-driven factor that protects endocrine-lineage cells against apoptosis in embryonic pancreas.

TFF2 was first identified in the pancreas of adult porcine^11^, and its expression has since been reported in several other organs including gastrointestinal tract^12,13^, immune cells^14–16^, lung^17,18^, kidney^19^ and skin^20^. Conflicting reports have prevented determination of the precise localization of TFF2 in the pancreas. Jackerott *et al*. demonstrated TFF2 mRNA expression in isolated human adult islets^21^, but that paper along with Madsen *et al*. reported no TFF2 protein expression in the acinar or islet cells of adult human pancreas^22^. In mice, Orime *et al*. showed TFF2 expression in adult β cells^4^, but Masui *et al*. reported by RNA-seq analyses that TFF2 expression was reduced in Rbpjl KO mice at E17.5^23^. Since Rbpjl is an indispensable compartment of the pancreas Transcription Factor 1 complex in acinar cells, they regarded TFF2 as an exocrine gene at this stage. We show in the present study by a more comprehensive analysis than previous works that TFF2 is expressed in exocrine cells but not in islet cells from E16.5. Seeing that our findings are consistent with the majority of previous papers, it would seem TFF2 protein is not expressed in adult islet cells.

A cell-protective function by TFF2 has been well documented in the stomach. TFF2 is expressed in the mucous neck cells of fundic glands and in the basal cells of the antral and pyloric glands and Brunner’s gland in duodenum^12,13^, and secreted TFF2 is believed to stabilize the mucous barrier that physically protects epithelial cells from acid-induced ulcerations^24^. TFF2 deficiency increases susceptibility to indomethacin-induced ulcerations in mice^25^, and the oral administration of TFF2 ameliorated ulcerations in TFF2 deficient rat^26^. However, because the subcutaneous injection of TFF2 also ameliorated ulcerations, it would seem this protein’s protective function is direct. Indeed, the anti-apoptotic effect of TFF2 was shown *in vitro* in several cancer cell lines: recombinant TFF2 reduced the apoptosis of MCF-7 and T47D breast carcinoma cell lines, and the addition of anti-TFF2 hSP3 accelerated the apoptosis of LS174T and SW480 colorectal carcinoma cell lines^27^. Here, we used explant culture experiments of embryonic pancreatic tissue to show that TFF2 prevents the apoptosis of embryonic Insulin+ cells through CXCR4. Furthermore, we showed that TFF2 exerts an anti-apoptotic effect on Nkx6.1+ trunk cells in Pdx1cKO mice, though the interacting receptor was unidentified. Thus, the hyper-activated apoptosis of endocrine lineage in Pdx1cKO mice can be explained by a lack of exocrine-driven TFF2. In addition, considering that TFF2 increases ERK1/2 phosphorylation and stimulates the proliferation of MIN6 cells and isolated adult murine islet^4^, TFF2 could be involved, at least in part, in the postnatal expansion of endocrine mass, which is extremely poor in the mutant mice^3^. At the same time, there exists endocrine defects in Pdx1cKO mice that cannot be explained by TFF2. For example, a previous study reported the numbers of Nkx6.1+ trunk cells, Ngn3+ endocrine precursors and Insulin+ cells are were reduced in the mutants as early as E14.5^3^, which is before the onset of TFF2 expression (E16.5). The identification of other exocrine-driven factors would deepen our understanding of how exocrine-endocrine interactions contribute to the development of endocrine pancreas.

## Methods

### Mice

To obtain Pdx1-conditional knock-out mice, Pdx1^loxP^ mice^28^ and Elastase-Cre mice^29^ were interbred as our previous report^3^. All animal experiments were performed in accordance with the Kyoto University guidelines for animal experiments and approved by the animal research committee of Kyoto University.

### Genotyping

DNA from the tail tips of mice was genotyped by PCR with the following primer sets: 1) Cre (forward) 5′-CGTACTGACGGTGGGAGAAT-3′ and (reverse) 5′-CCCGGCAAAACAGGTAGTTA-3′, product size 166 bp; and 2) Pdx1^loxP^ (forward) 5′-GGCTATCCACTCCTGCTCTG-3′ and (reverse) 5′-AGGTGGGTTCGCTAAACCTT-3′, product size 271 bp and 305 bp from WT and floxed alleles, respectively.

### Microarray analyses

Pancreata were dissected from postneonatal day 1 (control mice, n = 2, Pdx1cKO mice, n = 3) and immediately submerged in RNAlater (Ambion, Inc., Austin, TX). 250 ng of total RNA prepared with RNeasy Micro Kit (QIAGEN) was subjected to cDNA synthesis with GeneChip WT Expression Kit (Ambion), and the resultant cDNA was fragmented and hybridized to Mouse Gene 1.0 ST Array (Affymetrix). After hybridization, GeneChip arrays were washed and stained by GeneChip Fluidics Station 450 (Affymetrix) and detected by Scanner 3000 TG system (Affymetrix) according to the manufacturer’s instructions. Data analyses were performed with GeneSpringGX 12.1 software (Agilent Technologies). Microarray data have been deposited in Gene Expression Omnibus under accession number GSE118665.

### RNA isolation and RT-PCR

Total RNA was extracted using the RNeasy Mini Kit (Qiagen). First-strand cDNA synthesis was performed using Rever Tra Ace qPCR RT Master Mix (Toyobo). Quantitative RT-PCR was performed with SYBR Green. The primer sequences are listed in Supplementary Table S4. Independent experiments were performed three times and confirmed. Data were normalized in relation to the expression of GapDH. The analysis was performed using ΔΔCt methods.

### Tissue Preparation for Paraffin Sections

Tissue preparation and paraffin sections were performed as previously described^30^.

### Immunohistochemistry and immunofluorescence

Paraffin sections were cut into 4 μm thick slices. After rehydration, slides were washed and incubated for 30 min at room temperature with Protein Block (Dako) followed by overnight incubation at 4 °C with primary antibodies (Supplementary Table S2). The sections were washed in phosphate-buffered saline (PBS) and incubated for 60 min with secondary antibodies (Supplementary Table S3). Images were taken with a BZ-9000E HS All-in-one Fluorescence Microscope (Keyence).

### *In situ* hybridization

A digoxigenin-labeled RNA probe specific for murine *TFF2* was transcribed with digoxigenin-11-UTP according to the manufacturer’s instructions (Roche, Basel, Switzerland) by PCR using the primers 5′-CCTGCTGGCAGTGGTCCT-3′ and 5′-CAGACTGTGGGAAGAAACACC-3′. *In situ* hybridization was performed as described previously^31^. The alkaline phosphatase chromogen reaction was performed using Fast Red (Roche) as the substrate at room temperature for 48 hours. The sections were then washed with distilled water and mounted on glass slides in mounting medium. Control specimens for *in situ* hybridization (#SMPS-36, #SMPS-29) were obtained from 8 week-old C57BL/6 mice, which were purchased from GenoStaff (Tokyo, JPN).

### PHH3 staining and TUNEL assays

Mitotic activity was analyzed by immunolabeling with rabbit anti-phospho-Histone H3 (Ser10) (Millipore). TUNEL assays were done using the DeadEnd Fluorometric system (Promega) according to the manufacturer’s instructions.

### Explant culture

We obtained pancreata explant from control and Pdx1cKO mice at E16.5. Pancreata explants were cultured in DMEM/Ham F-12 (Invitrogen) containing 1% L-glutamine, 10% fetal bovine serum (FBS) and 0.5% penicillin-streptomycin (Invitrogen). Pancreata explants were cultured with a 24-well Ultra Low Attachment Surface plate (Costar) for 48 hours.

### Reagents

Recombinant mouse TFF2 (#RPA748MU01) was purchased from Uscn Life Science (Texas, USA). AMD3100 (#AB120718) was purchased from Abcam (Cambridge, GBD).

### Cell Counting

To evaluate cell numbers in pancreas tissue at E16.5, the whole pancreas was sectioned in 4 μm intervals, and the number of Insulin+ cells was counted every 150 μm. To evaluate the cell numbers on pancreata explant *in vitro*, the whole explant was sectioned in 4 μm intervals, and the number of Insulin- or Nkx6.1-positive cells was counted every 100 μm. Microscopic observation was performed at 200x magnification.

### Statistical analysis

All statistical analyses were performed using Student’s t-tests or one-way analysis of variance (ANOVA) implemented in JMP Pro 14 Software (SAS Institute, Cary, NC, USA) to evaluate differences between groups. A value of P < 0.05 was considered to indicate a statistically significant difference.

## Supplementary information

Supplementary Information
