# Supplementary material for: Exocrine tissue-driven TFF2 prevents apoptotic cell death of endocrine lineage during pancreas organogenesis
Source: Sci Rep. 2019 Feb 7;9:1636. doi: 10.1038/s41598-018-38062-9 (PMC6367380; doi:10.1038/s41598-018-38062-9)
Supplement: Supplementary file 1 — Supplementary Information [file 41598_2018_38062_MOESM1_ESM.pdf]

Supplementary Information

Exocrine tissue-driven TFF2 prevents apoptotic cell death of endocrine lineage during pancreas organogenesis

Koji Hirata, Sota Kodama, Yasuhiro Nakano, Yasuko Minaki-Nakagawa, Yoshiki Aoyama, Morito Sakikubo, Toshihiko Goto, Masahiro Yoshida, Toshihiko Masui, Takuya Yamamoto, Shinji Uemoto and Yoshiya Kawaguchi

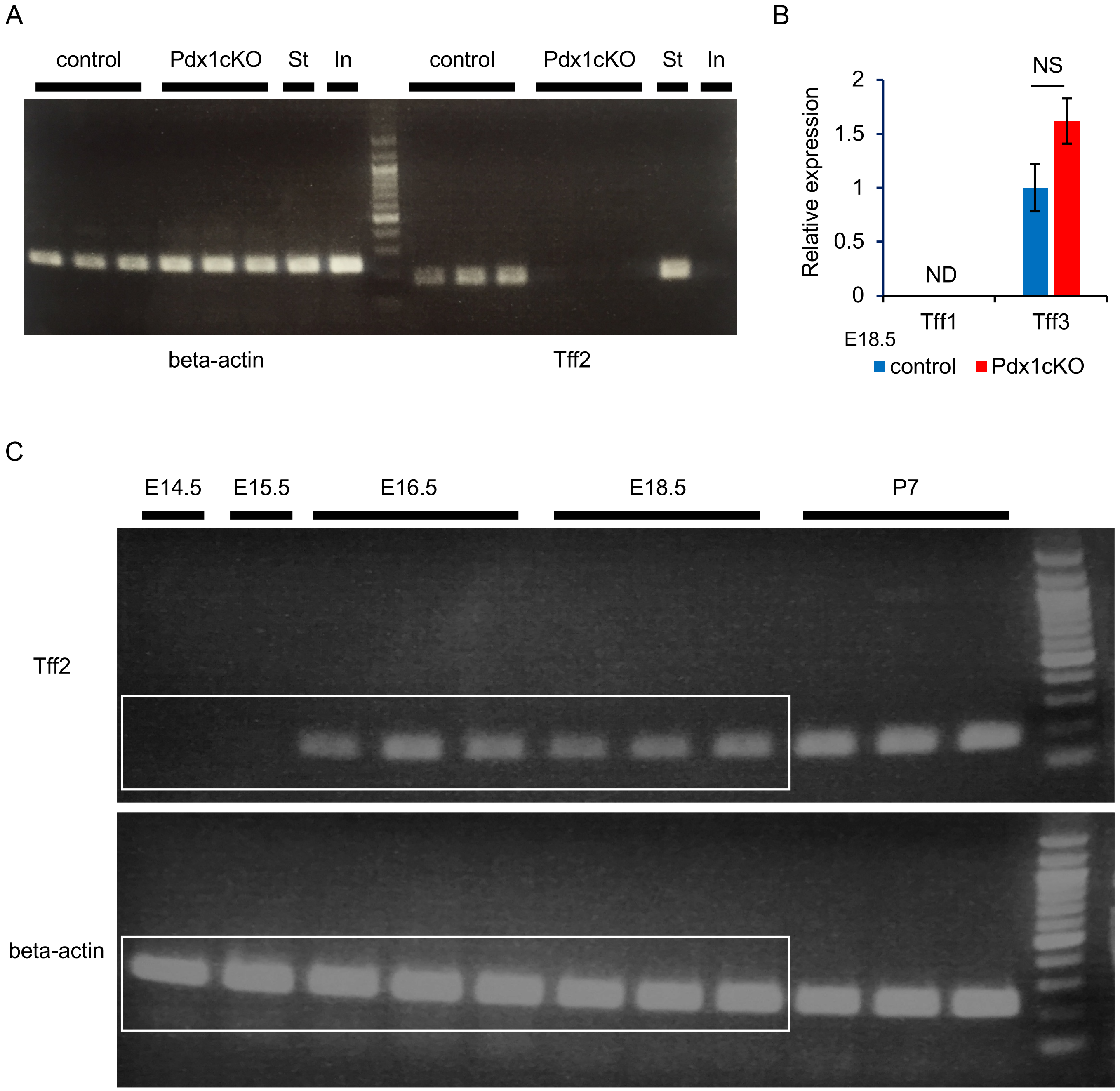

Supplementary Figure S1. The expression of *TFF* family in mice pancreas by RT-PCR.

(A) The expression of *TFF2* mRNA detected by RT-PCR at P1 in control and Pdx1cKO mouse pancreata. St: stomach of WT mice (positive control), In: small intestine of WT mice (negative control). (B) The relative expressions of *TFF1* and *TFF3* mRNA at P1 in both pancreata. (C) Original RT-PCR data of Figure 1A. Approximate regions used for figures are marked with rectangles. The ladder shows markers every 100 bp. The bottom band is 100 bp. Bars represent the mean $\pm$ SE.

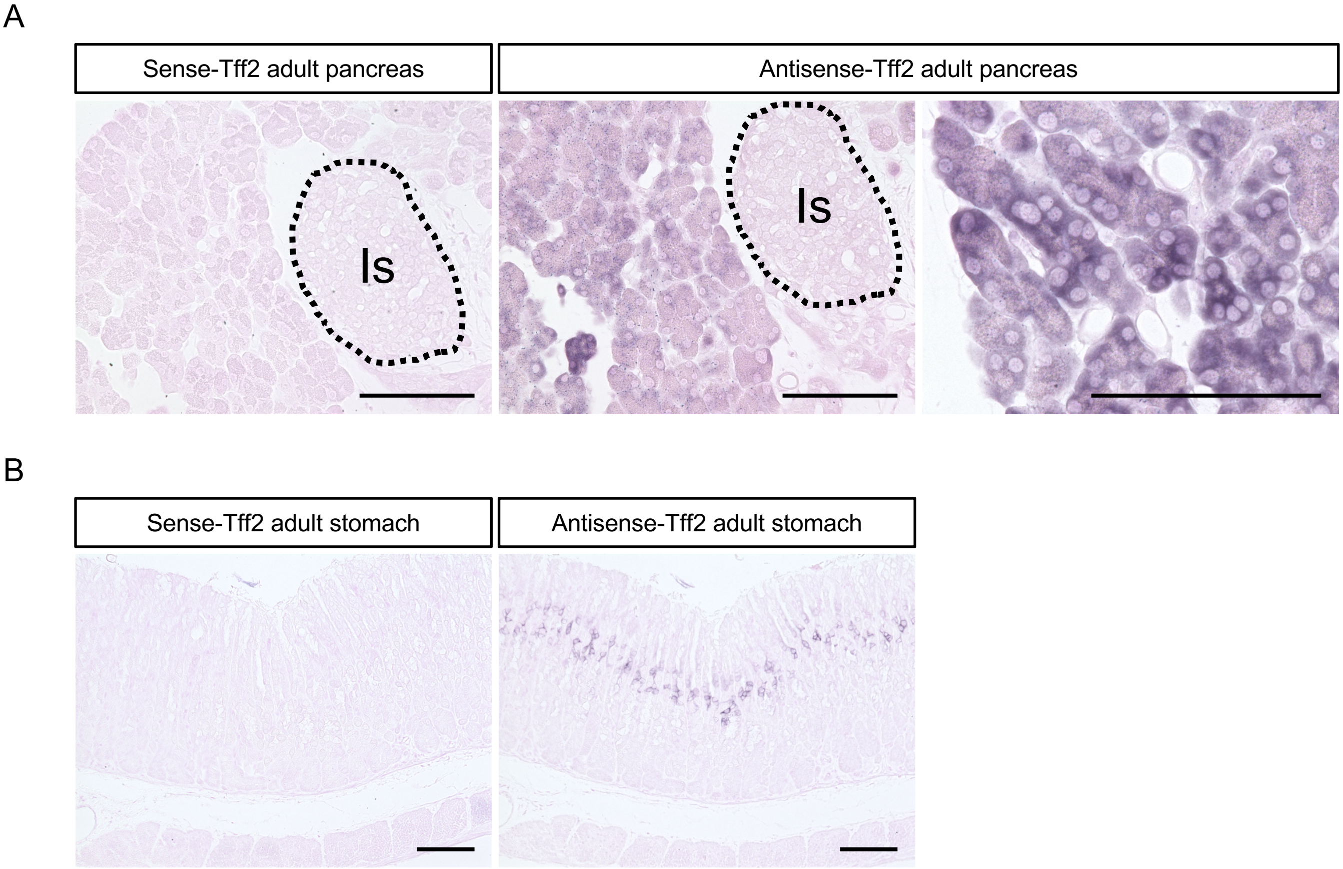

Supplementary Figure S2. Localization of *TFF2* mRNA in control adult mice.

The localization of endogenous *TFF2* by in situ hybridization on sections of pancreas and stomach in control mice. (A) *TFF2* mRNA is detected in acinar of adult mice pancreas (middle and right panel) by an anti-sense probe. *TFF2* mRNA expression is hardly observed in adult mice islet. Left panel shows negative control with a sense probe. Is: Islet. (B) *TFF2* mRNA was detected in mucous neck cells of adult mice stomach (right panel). Left panel shows negative control with a sense probe. Scale bars, 100 µm.

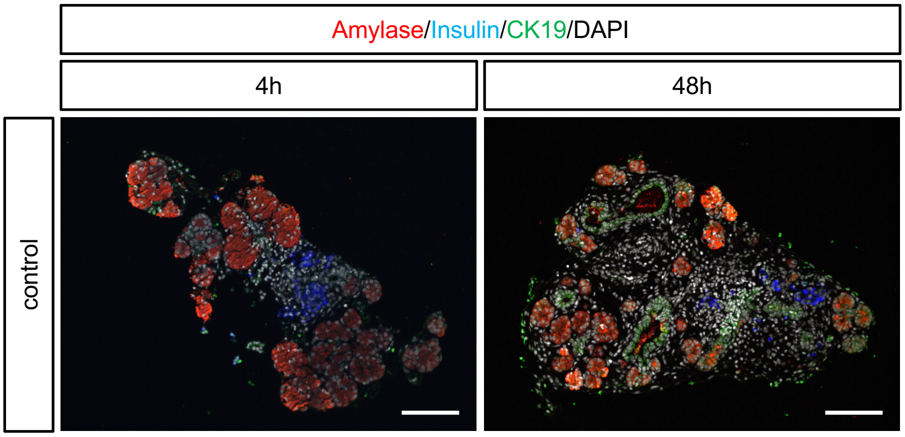

Supplementary Figure S3. Explant culture with conditioned medium in E16.5 control mice.

Immunostaining of amylase (red), insulin (blue), CK19 (green) and DAPI (white) in control mice pancreatic explant at 4 hours after (left panel) and 48 hours after (right panel). Pancreatic explant shows similar compositions at 4 hours and 48 hours. The percentage of pHH3 positive cells among insulin-expressing cells tended to decrease on cultured explant at 48 hours compared with in vivo pancreas at E16.5. Scale bars, 100 µm.

Transcripts Cluster ID	Fold Change	Regulation	genesymbol	genedescription
10581355	48.523033	down	Ctrl	chymotrypsin-like
10464328	42.33804	down	Pnliprp2	pancreatic lipase-related protein 2
10551531	33.378933	down	Sycn	syncollin
10432652	24.593367	down	Cela1	chymotrypsin-like elastase family, member 1
10498741	24.02757	down	Serpini2	serine (or cysteine) peptidase inhibitor, clade I, member 2
**10449661**	**21.991234**	**down**	**Tff2**	**trefoil factor 2 (spasmolytic protein 1)**
10568165	18.505526	down	Zg16	zymogen granule protein 16
10558159	18.343609	down	Dmbt1	deleted in malignant brain tumors 1
10458704	11.441293	down	Spink3	serine peptidase inhibitor, Kazal type 3
10544338	11.2995825	down	Prss3	protease, serine, 3
10539795	10.904543	down	Gkn2	gastrokine 2
10466027	10.085808	down	Pga5	pepsinogen 5, group I
10390850	9.687266	down	Krt20	keratin 20

Supplementary Table S1. Secreted factors and acinar-associated genes downregulated by Pdx1 inactivation.

Supplementary Table S2. Primary Antibodies

Antigen	Species	Dilution	Supplier (Cat.#)
TFF2	Rabbit	1:50	Proteintech, Rosemont, IL, USA (13681-1-AP)
CXCR4	Goat	1:300	Capralogics Inc., Hardwick, MA, USA (CI0116)
Insulin	Guinea Pig	1:400	Dako, Glostrup, DNK (A0564)
Nkx6.1	Mouse	1:300	DSHB, Iowa, IA, USA (F55A12)
beta-catenin	Mouse	1:300	BD Biosciences, Franklin Lakes, NJ, USA (610154)
Amylase	Rabbit	1:1000	Sigma, Tokyo, JPN (A8273)
CK19	Goat	1:100	Santa Cruz, Dallas, TX, USA (sc-33111)
PHH3	Rabbit	1:1000	Millipore, Burlington, MA, USA (04-1093)

Supplementary Table S3. Secondary Antibodies

Conjugate	Antigen	Species	Dilution	Supplier (Cat.#)
Allexa Fluor 488	Rabbit IgG	Donkey	1:1000	Molecular Probes, California, USA
Allexa Fluor 488	Mouse IgG	Donkey	1:1000	Molecular Probes, California, USA
Allexa Fluor 488	Goat IgG	Donkey	1:1000	Molecular Probes, California, USA
Cy3	Guinea Pig IgG	Donkey	1:400	Chemicon, Massachusetts, USA
Cy3	Rabbit IgG	Donkey	1:400	Chemicon, Massachusetts, USA
Cy3	Mouse IgG	Donkey	1:400	Chemicon, Massachusetts, USA
Cy5	Guinea Pig IgG	Donkey	1:400	Chemicon, Massachusetts, USA
HRP(ImmuPress)	Rabbit IgG	Horse	1:1	Vector Laboratories, California, USA

Supplementary Table S4. RT-PCR primer sets

Gene		Sequence
GapDH	Forward	5'-GTGTTCCTACCCCCAATGTGT-3'
	Reverse	5'-ATTGTCATACCAGGAAATGAGCTT-3'
beta-actin	Forward	5'-AGCCATGTACGTAGCCATCC-3'
	Reverse	5'-CTCTCAGCTGTGGTGGTGAA-3'
Tff1	Forward	5'-ATCTGTGTCCTCGCTGTGGT-3'
	Reverse	5'-CCGGACACTGTCATCAAAAC-3'
Tff2	Forward	5'-TGCTTTGATCTTGGATGCTG-3'
	Reverse	5'-GGAAAAGCAGCAGTTTCGAC-3'
Tff3	Forward	5'-CTGGTTGCTGGGTCCTCTG-3'
	Reverse	5'-GGGCACATTTGGGATACTGG-3'
